# Chronic Pulmonary Aspergillosis During Convalescence From Severe COVID-19 Treated With Oral Itraconazole: A Report of Two Cases

**DOI:** 10.7759/cureus.27281

**Published:** 2022-07-26

**Authors:** Hiroshi Horiuchi, Syusuke Utada, Yoshie Shinomiya, Takao Miyagawa, Azusa Sogo, Shoko Niida, Hiromu Okano, Naoya Suzuki, Tsuyoshi Otsuka, Hiroshi Miyazaki, Ryosuke Furuya

**Affiliations:** 1 Department of Critical Care and Emergency Medicine, National Hospital Organization Yokohama Medical Center, Yokohama, JPN

**Keywords:** lung cavity, itraconazole, chronic pulmonary aspergillosis, aspergillus, covid-19

## Abstract

Invasive pulmonary aspergillosis (IPA) has been reported to occur secondary to coronavirus disease 2019 (COVID-19), and the condition has been termed COVID-19-associated pulmonary aspergillosis (CAPA). We diagnosed two severe COVID-19 cases with multiple cavitary lung lesions and chronic pulmonary aspergillosis (CPA) on days 58 and 48 of admission, respectively, with gradual improvement in the respiratory status. Both patients were positive for *Aspergillus-*precipitating antibodies (APAb). We chose oral itraconazole (ITCZ) for both patients because of its convenience in terms of long-term treatment. Cavitary lesions diminished after ITCZ administration. The risk factors for pulmonary aspergillosis in both patients were determined to be steroid pulse therapy, use of baricitinib, diabetes mellitus (DM), ICU admission, long hospital stay, and the use of broad-spectrum antibiotics. Pulmonary aspergillosis must be suspected in patients with severe COVID-19, even if they are asymptomatic, because not only IPA but also CPA can occur following COVID-19. Therefore, oral ITCZ may be a treatment option for CPA following COVID-19.

## Introduction

Invasive pulmonary aspergillosis (IPA) has been reported in patients secondary to coronavirus disease 2019 (COVID-19), especially in those with severe disease requiring invasive mechanical ventilation (IMV) [1]. This phenomenon has been recognized as COVID-19-associated pulmonary aspergillosis (CAPA), for which the incidence rate and mortality have been reported to be as high as 13.5% and 48.4%, respectively [1]. The recommended first-line therapy is voriconazole (VRCZ) or isavuconazole, similar to treatment for IPA in non-COVID-19 patients [2]. However, chronic pulmonary aspergillosis (CPA) associated with COVID-19 has been rarely reported. Compared to the severity of IPA, some patients with CPA are asymptomatic and identified radiologically. *Aspergillus* IgG antibody in the blood is usually required for the diagnosis of CPA. We discuss two cases of CPA that developed during convalescence from severe COVID-19. Both patients presented with multiple cavitary lung lesions that diminished in size following oral administration of itraconazole (ITCZ).

## Case presentation

Case 1

**Figure 1:**
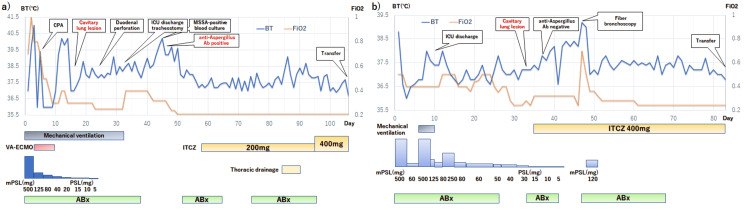
Clinical course based on body temperature (BT) and fraction of inspired oxygen (FiO2) a) Case 1, b) Case 2 When oxygen was supplied by a face mask or nasal cannula, FiO_2_ was calculated according to the normal calibration scale CPA: cardiopulmonary arrest; Day: day since admission; Abx: antibiotics; ITCZ: itraconazole; mPSL: methylprednisolone; PSL: prednisolone

**Figure 2:**
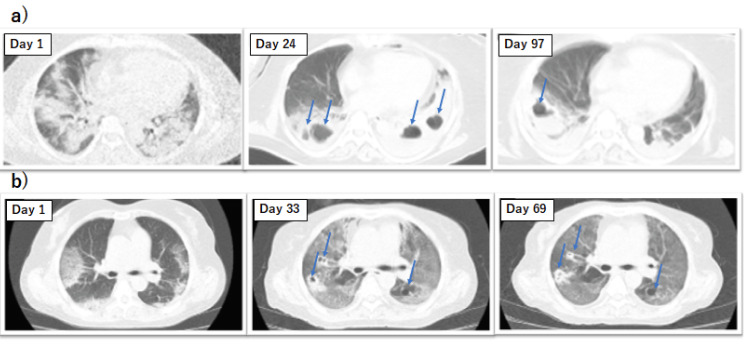
Serial chest CT findings a) Case 1: the bilateral cavitary lesions (arrows), one of which was recognized by X-ray on day 16, were recognized by CT on day 24. After the administration of ITCZ from day 58, the cavitary lesions diminished on day 97 b) Case 2: the bilateral cavitary lesions (arrows) were recognized for the first time on day 33. After the administration of ITCZ from day 35, the cavitary lesions diminished on day 69 CT: computed tomography; ITCZ: itraconazole

A 49-year-old woman with diabetes mellitus (DM), hypertension, hyperuricemia, and obesity with a BMI of 33.8 was admitted to our hospital due to worsening dyspnea and a non-productive cough lasting six days. She had tested positive for severe acute respiratory syndrome coronavirus 2 (SARS-CoV-2) via a PCR test two days ago. Upon arrival, she was dyspneic and hypoxic. She had an oxygen saturation level of approximately 90% under oxygen therapy at 10 L/minute. A CT scan showed widespread bilateral ground-glass opacities. She was intubated and admitted to the ICU (day one). Drug therapy with methylprednisolone (mPSL) pulse was initiated, which was tapered every three days (Figure 1a). Venoarterial extracorporeal membrane oxygenation (VA-ECMO), which was started on day three due to pulmonary embolism (PE), was withdrawn on day four but was restarted on day seven following cardiopulmonary arrest suspected to be caused by PE. Although she could be withdrawn again from VA-ECMO on day nine, a CT scan showed findings of hypoxic-ischemic encephalopathy, and her level of consciousness did not improve. Although her respiratory condition gradually improved, a routine chest X-ray follow-up showed a suspected cavitary lesion in the right lower lung field on day 16. On day 23, minor duodenal perforation was observed, and conservative therapy was administered. Multiple cavitary lesions were observed in both lungs on the same day (Figure 2a). A tracheostomy was performed, and the patient was discharged from the ICU on day 32.

On day 45, body temperature (BT) increased to 40.2 ºC, and blood cultures were taken. Although her respiratory condition did not worsen, a gradual increase in the cavitary lesion in the right lower lung field and right pleural effusion were observed. Pleurocentesis revealed bloody exudative effusion, and bacterial and fungal cultures were negative. Because 1,3-beta-D-glucan (BDG) and *Aspergillus* antigen galactomannan (AAg) levels on day 42 were 124 pg/mL and 0.6 (cut-off index: 0.5), respectively, *Aspergillus-*precipitating antibody (APAb) was measured on day 46 (Table 1a). BT decreased gradually without the use of antibiotics until day 52, while one set of blood cultures on day 45 was positive for methicillin-resistant *Staphylococcus aureus* (MRSA). Cefazolin 2 g q8h intravenous (IV) was started, and blood cultures were taken on day 52. On day 58, APAb taken on day 46 was reported to be positive, and ITCZ 200 mg per ou (p.o.) was initiated based on the diagnosis of CPA. Blood cultures on day 52 were negative, and cefazolin was continued until day 65. Thoracic drainage was performed from day 84 to day 90 due to the increase in the right pleural effusion and fever up to 39 ºC despite the use of piperacillin-tazobactam (TAZ/PIPC) 4.5 g q6h IV from day 73. Bacterial and fungal cultures of the pleural effusion were negative. BT decreased after we increased the dose of ITCZ to 400 mg p.o. on day 95, and CT findings on day 97 revealed a decrease in the size of cavitary lesions in both lungs. The patient was transferred to a long-term care facility because of a disturbance of consciousness on day 106.

Case 2

A 62-year-old woman with a history of Ménière's disease was transferred to our hospital on account of worsening fever and dyspnea three days prior; she had tested positive for SARS-CoV-2 in a PCR test performed on that day. Upon arrival, she was dyspneic and hypoxic. She had oxygen saturation levels of approximately 95% under oxygen therapy at 6 L/minute. The CT scan showed widespread bilateral ground-glass opacities and consolidation superior to the dorsal lungs. High-flow nasal cannula (HFNC) oxygen delivery (50 L/minute with FiO_2_:0.50) was initiated. Drug therapy, including a half mPSL pulse, was initiated (Figure 1b). On days 6-10, she was on mechanical ventilation in the ICU due to constrained respiration and worsening CT findings. On day 11, she was discharged from the ICU to the emergency ward. The mPSL was tapered. We continued to give TAZ/PIPC 4.5 g q6h IV until day 29 because of nosocomial pneumonia and the detection of *Serratia marcescens* in her sputum on day 12. The CT scan performed due to elevation of the inflammatory response on day 33 revealed multiple cavitary lung lesions bilaterally (Figure 2b). We restarted TAZ/PIPC 4.5 g q6h IV on day 33, which seemed ineffective until day 35. On day 35, we started ITCZ 400 mg p.o., suspecting pulmonary aspergillosis. The BDG and AAg levels on day 33 were 821 pg/ml and 0.1, respectively. The APAb measured on day 37 was negative (Table 1b).

To confirm the diagnosis, we performed fiber bronchoscopy on day 47 and detected septate filamentous fungi, consistent with *Aspergillus* in the bronchoalveolar lavage (BAL) and endobronchial biopsy samples. Fever up to 39.0 ºC, increased oxygen demand, and wheezing were noted on day 48. We started TAZ/PIPC 4.5 g q6h IV and mPSL 40 mg q8h IV, considering aspiration pneumonia induced by bronchoscopy aggravated by an asthma attack. Although her APAb test result was negative, CPA was diagnosed. The reasons for this are as follows: (1) filamentous fungi consistent with *Aspergillus* were detected in the BAL and histopathological samples of the bronchus; (2) her clinical symptoms related to respiration were not prominent; and (3) the AAg levels were not elevated. We de-escalated TAZ/PIPC to cefozopran 2 g q12h IV and continued it until day 67 because we detected *Serratia marcescens* in the BAL on day 47. Her respiratory status gradually recovered, and CT images on day 69 showed shrinkage of the cavitary lesions in both lungs. Her oxygen demand was maintained at 1 L/minute on exertion. The patient was transferred to a long-term care hospital for rehabilitation on day 83. The APAb measured on day 173 returned positive on an outpatient follow-up basis.

Figure 1 depicts the clinical course of two patients in terms of BT and FiO_2_, while Figure 2 shows the serial CT findings.

Table 1 lays out the dynamics of serum markers of aspergillosis in both cases.

**Table 1:** The dynamics of serum markers of aspergillosis a) Case 1, b) Case 2 The normal values are as follows: BDG: <20.0 pg/ml; AAg: <0.5; APAb: negative BDG: 1,3-beta-D-glucan; AAg: *Aspergillus* antigen galactomannan; APAb: *Aspergillus-*precipitating antibody; NA: not assessed

a)											
Day	7	11	15	18	23	28	35	42	46	49	95
BDG (pg/ml)	NA	39	NA	50	49	52	102	124	NA	95	NA
AAg	0.6	NA	0.5	NA	NA	NA	0.6	0.6	NA	0.8	0.2
APAb									Positive		

b)								
Day	11	20	25	33	37	46	73	173
BDG (pg/ml)	29	293	272	821	NA	176	102	NA
AAg	0.3	0.1	0.1	0.1	NA	NA	0.2	NA
APAb					Negative	NA		Positive

## Discussion

These cases illustrate two major clinical issues. Firstly, we showed that CPA can occur following a diagnosis of COVID-19. Second, ITCZ, which is not the first treatment of choice for pulmonary aspergillosis, can be effective for the treatment of CPA following COVID-19.

In Case 2, although aspergillosis was suggested by cytological and histological findings, the BAL culture did not show positive results for any fungi on day 47. We believe this was due to the prior administration of ITCZ from day 35. Although infections of filamentous fungi such as *Fusarium*, *Scedosporium*, *Trichosporon*, and *Mucor* were included cytologically in the differential diagnosis, the effectiveness of ITCZ and the elevation of BDG did not indicate infections due to these fungi. We initiated TAZ/PIPC from day 33, which indicated that the elevation of BDG on day 33 was not due to the administration of TAZ/PIPC. We could not identify any other causes of the false positivity of BDG. We did not find a positive result for APAb on day 37, which may be partly due to the continuous administration of steroids from day one. On day 37, 30 mg of prednisolone was administered. In addition, we might have measured APAb too early because anti-*Aspergillus* antibody production has been reported to start from a mean of 10.8 days after the onset of infection in immunosuppressed patients [3]. Finally, the APAb positivity on day 173 confirmed the diagnosis.

The patients in this study were diagnosed with CPA. The time to CAPA diagnosis from ICU admission and IMV initiation has been reported to range between 4.0-15.0 days and 3.0-8.0 days [1]. In Case 1, a cavitary lung lesion first appeared on day 16, which was 16 days after ICU admission and 16 days after the initiation of mechanical ventilation. We did not suspect IPA when we first recognized the cavitary lung lesion on day 16 because severe symptoms related to IPA were not obvious at that time, and the patient's respiratory status was gradually improving. Since the lung cavitary lesions did not progress quickly, IPA was not suspected, and the diagnosis of pulmonary aspergillosis was delayed until day 58. In Case 2, a cavitary lung lesion first appeared on day 33, 27 days after ICU admission, and 27 days after the initiation of mechanical ventilation. On day 33, as in Case 1, severe symptoms related to IPA were not obvious, and the patient's respiratory status was gradually improving. Although the definition of CAPA is still unclear, one article describes that “CAPA is defined as IPA in temporal proximity to a preceding SARS-CoV-2 infection” [1]. Based on this definition, our cases were not classified as CAPA. A case of chronic cavitary pulmonary aspergillosis (CCPA) after COVID-19 has been reported, but this case was quite different from ours because in this case latent aspergilloma was considered to be activated after SARS-CoV-2 infection [4]. CCPA is characterized by symptoms lasting for at least three months [5,6], and our cases did not meet this criterion even at the time of transfer to the long-term care hospital. We believe that our cases had subacute invasive pulmonary aspergillosis (SIPA), formerly known as chronic necrotizing pulmonary aspergillosis, which progresses over a period of one to three months. Both of our cases did not have severe symptoms related to IPA when lung cavitary lesions were first recognized, which was the main reason we diagnosed our cases as CPA, not IPA.

ITCZ, which is not the first treatment of choice for pulmonary aspergillosis, can be effective in the treatment of CPA following COVID-19. VRCZ is usually the first choice for treating CPA, as well as IPA [7]. It is also preferred for treating SIPA [8,9]. We treated our patients with oral ITCZ, but not VRCZ, for the following reasons: (1) both patients had to be transferred to chronic hospitals, and oral ITCZ is more widely available in Japan than VRCZ; (2) oral ITCZ is less expensive than oral VRCZ; and (3) less adverse effects have been reported with ITCZ compared to VRCZ [10]. The most frequent adverse effect of ITCZ is cardiotoxicity, and neither patient had any cardiovascular problems. Although we treated Case 1 with oral ITCZ 200 mg once daily from day 58 to day 94 and increased the dose to 200 mg twice daily on day 95 due to the continuous fever, the CT findings on day 97 indicated that the administration of ITCZ 200 mg once daily was effective. Therapeutic dose monitoring for ITCZ is not commercially available. We treated Case 2 with oral ITCZ 200 mg twice daily from day 35, and the CT findings on day 69 showed the effectiveness of the treatment. One of the limitations of this report is that we could follow the clinical courses in both cases for only less than two months and CPA usually requires treatment for at least six months [11]. One network-centric meta-analysis has recently reported that ITCZ is the best oral antifungal agent for the initial treatment of CPA [12]. Further studies on CPA treatment following COVID-19 are required.

Classically, risk factors for IPA include severe and prolonged neutropenia, receiving high doses of glucocorticoids, and cellular immune dysfunction status. For non-COVID-19-associated CPA, independent risk factors related to mortality were reported to include prolonged hospital stay, hematological malignancies, lung consolidation, and ICU admission; chronic kidney disease and DM have also been associated with poor outcomes [13]. Although many uncertainties regarding CPA remain, one review article has reported that prior treatment with broad-spectrum antibiotics such as meropenem (MEPM) and TAZ/PIPC might be a risk factor for the development of CAPA [14]. Prior to the appearance of lung cavities in both of our cases, they had been treated with steroid pulse therapy and baricitinib for COVID-19, and MEPM or TAZ/PIPC was administered for ventilator-associated pneumonia. Neither of the patients experienced neutropenia during their clinical course. Case 1 had pre-existing DM, whereas Case 2 was diagnosed with DM after admission. DM is known to be one of the major risk factors for COVID-19. A meta-analysis has shown that the pooled prevalence of DM in patients with COVID-19 was 16.8%, and patients with DM had a significantly higher mortality rate compared to COVID-19 patients without DM (22.14% vs. 12.81%) [15]. Patients with severe COVID-19 who are more likely to have DM are usually treated with immunosuppressants such as steroids, baricitinib, and tocilizumab in the ICU. While they are on mechanical ventilation, they are often treated with broad-spectrum antibiotics for bacterial infections, such as ventilator-associated pneumonia and catheter-related bloodstream infections. Patients with severe COVID-19 tend to have longer hospital stays, partly because they are often elderly. Although the definition of CAPA is still unclear, pulmonary aspergillosis should be suspected in all patients with severe COVID-19 because of its high incidence rate and high mortality.

One of the limitations of this study was that the identification of *Aspergillus* species and antifungal sensitivities could not be reported since fungal cultures were negative. Another was that the optimal duration of ITCZ therapy was unknown. Because both cases were transferred to a long-term care hospital, administration of ITCZ has been continued in both cases, assuming the need for lifelong antifungal treatment.

## Conclusions

Pulmonary aspergillosis must be suspected in patients with severe COVID-19, even if they are asymptomatic, because not only IPA but also CPA can occur following COVID-19. For COVID-19 patients with a prolonged hospital stay, we recommend performing chest radiography regularly at least once a week. If cavitary lung lesions are observed, screening for pulmonary aspergillosis is desirable even if they do not seem to be related to any symptoms. Furthermore, oral ITCZ may be one of the best treatment options for these patients. In the future, changes in the definition of CAPA might lead to CPA cases being included under its purview. Since only two years have passed since the emergence of the COVID-19 pandemic, studies with longer follow-up durations are needed and better treatment options must be explored.
